# Isolated Gastric Myeloid Sarcoma: A Case Report and Review of the Literature

**DOI:** 10.1155/2014/541807

**Published:** 2014-07-06

**Authors:** Pankit Vachhani, Prithviraj Bose

**Affiliations:** ^1^Department of Internal Medicine, Virginia Commonwealth University (VCU), P.O. Box 980663, 1001 E Broad Street, Old City Hall, Suite 405, Richmond, VA 23298, USA; ^2^Massey Cancer Center, Virginia Commonwealth University (VCU), P.O. Box 980070, 1201 E Marshall Street, MMEC 11-213, Richmond, VA 23298, USA

## Abstract

Myeloid sarcoma represents the proliferation of myeloblasts of acute myeloid leukemia (AML) at extramedullary sites. While extramedullary involvement in AML is uncommon in itself, isolated myeloid sarcomas, that is, myeloid sarcomas without any bone marrow involvement, are extremely rare and pose a diagnostic and therapeutic challenge. Here, we present the case of a middle-aged woman with isolated myeloid sarcoma in the stomach—an organ seldom involved by this disease. Additionally, the literature on the epidemiology, diagnosis, pathology, prognosis, and therapeutic options in myeloid sarcomas has been reviewed.

## 1. Introduction

Acute myeloid leukemia (AML) is a cancer of the myeloid elements of the bone marrow characterized by the rapid proliferation of abnormal blasts in the bone marrow that interfere with normal hematopoiesis. Rarely, however, the disease can manifest with extramedullary organ involvement, known as myeloid sarcoma (MS) or, previously, chloroma or granulocytic sarcoma. Defined as the presence of proliferating myeloid blasts in an extramedullary site that disrupts the normal architecture of the organ in which they are found [1], MS is recognized as a distinct entity under AML and related myeloid neoplasms in the 2008 World Health Organization classification [2]. While commonly associated with AML, MS has rarely been described in association with other myeloid neoplasms, including myelodysplastic syndrome and myeloproliferative neoplasms [1, 3, 4]. MS can occur following, concurrently with, or preceding bone marrow involvement of AML. Isolated MS, also known as primary or* de novo* MS, represents MS without any blood or bone marrow involvement at the time of diagnosis. This entity can involve almost any organ system, including the skin (“leukemia cutis”), lymph nodes, bone, brain, breast, cervix, and visceral organs. Here, we present the case of a middle-aged woman who was found to have isolated MS in the stomach, a very infrequent site of involvement.

## 2. Case Presentation

**Figure 1:**
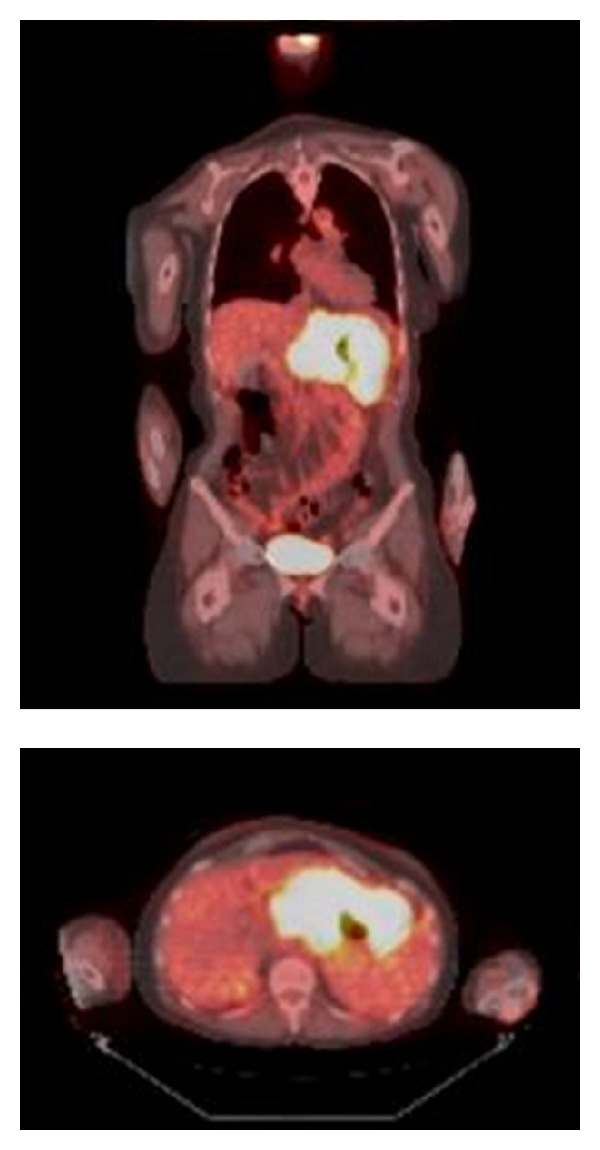
Coronal and cross-sectional PET/CT images demonstrate intense radiotracer uptake in the gastric region.

A 52-year-old otherwise healthy female presented with a month-long history of dyspepsia. A trial of proton-pump inhibitor therapy proved unsuccessful and she developed severe dysphagia and odynophagia over the ensuing three months. Examination was unremarkable besides epigastric tenderness. Computed tomography (CT) revealed 3-cm gastric wall thickening, most prominent in the fundus and body, extending into the gastrohepatic ligament and along the celiac artery, inseparable from the pancreatic body and tail. Esophagogastroduodenoscopy (EGD) showed a gastric fundus mass and pathology revealed infiltration of the lamina propria with large atypical cells that demonstrated nuclear pleomorphism with prominent nucleoli. Immunohistochemistry (IHC) was positive for CD4, CD33, CD34, CD43, CD45, CD68, CD117, CD163, and lysozyme. The neoplastic cells were, however, negative for CD3, CD5, CD8, CD20, CD30, CD56, CD138, PAX5, cyclin D1, MUM-1, BCL-6, pan-keratin AE1/3, TIA, myeloperoxidase, and EBER (Epstein Barr encoded RNA) by ISH (*in situ* hybridization). CD10, CD128, TdT, and BCL-2 staining were variably positive, and the proliferation fraction (Ki-67) was 60%. However, morphologic bone marrow examination, IHC, karyotyping, and FISH (fluorescence* in situ* hybridization) using probes designed to detect rearrangements of the* c-MYC *(8q24),* CDKN2A* (9p21), and* MLL* (11q23) loci as well as t(9;22) and t(12;21) were normal. Complete blood count (CBC) revealed leukocytes of 3,000/*μ*L, hemoglobin of 11.8 g/dL, and platelets of 201,000/*μ*L; the blood smear showed no circulating blasts. A diagnosis of isolated gastric MS, monocytic subtype (by the French American British classification), was made based on the expression of CD34, CD117 (both blast markers), CD4, CD68, CD163, and lysozyme (suggestive of monocytic differentiation). Positron emission tomography with CT (PET/CT) showed intense and diffuse radiotracer uptake in the thickened gastric wall with a maximum standardized uptake value (SUV_max⁡_) of 14.7 that was closely associated with adjacent liver, pancreas, and spleen (Figure 1). Induction chemotherapy with standard-dose cytarabine and high-dose daunorubicin [5] was begun and she improved symptomatically. Follow-up EGD-guided gastric biopsy showed continued presence of MS. Similarly, follow-up PET/CT showed persistent radiotracer uptake with SUV_max⁡_ of 11.2 in the gastric wall and perigastric region. Repeat bone marrow biopsy showed no evidence of malignancy. Reinduction chemotherapy with fludarabine, cytarabine, idarubicin, and filgrastim was initiated. Subsequent PET/CT showed persistent radiotracer uptake (SUV_max⁡_ 12.2) and slight decrease in size of the mass. However, EGD-guided gastric biopsy revealed no malignant cells. Immunophenotyping was unremarkable as well. Given the PET/CT findings, however, it was thought that the biopsies had missed malignant sites. Surgical resection was not recommended considering the involvement of adjacent structures. Radiation therapy to a total dose of 27 Gy over 15 fractions was administered. Significant reduction of metabolic activity in the gastric region (SUV_max⁡_ 6.2) was noted on a follow-up PET/CT scan. Three months later, the patient presented with abdominal pain and vomiting. CBC was normal, but CT scan showed peritoneal carcinomatosis. Fine needle aspirate of a pelvic lymph node was consistent with the previously rendered diagnosis of MS. At this point, the patient decided to forego active treatment and died a few weeks later.

## 3. Discussion

The rarity of MS is reflected by its incidence of 2–9% of all AML cases [6–8]. Isolated MS is even rarer; its true incidence is probably unknown. Cases of gastric MS arising in the context of previously diagnosed hematologic neoplasms have been described in the literature [9–13]. However, isolated gastric MS is an exceedingly rare diagnosis. In a comprehensive review of the literature spanning almost 30 years, only 2 of 154 reported cases of isolated MS involved the stomach [3]. To our knowledge, there exist only two other documented cases [14, 15].

In nearly half the cases (47%), MS is initially misdiagnosed, with lymphoma being the most common incorrect diagnosis [3, 16]. Indeed, on light microscopy, the differential diagnosis of MS includes non-Hodgkin's lymphoma, lymphoblastic leukemia, melanoma, Ewing's sarcoma, primitive neuroectodermal tumor, rhabdomyosarcoma, neuroblastoma, medulloblastoma, undifferentiated carcinoma, blastic plasmacytoid dendritic cell neoplasm, and extramedullary hematopoiesis [8, 17, 18]. Further diagnostic studies in the evaluation of MS should include IHC, flow cytometry, cytogenetic studies, and molecular and genetic mutation analysis on both the malignant mass and bone marrow to evaluate for low level marrow involvement in the absence of gross disease. CT or PET/CT can be used to image the tumor and monitor response to treatment [8].

Previous studies had indicated that the core-binding factor (CBF) abnormality t(8:21) was the most common cytogenetic abnormality associated with MS formation [19, 20]. However, in an Italian study of 92 MS cases, t(8:21) was rare (2.2%), while monosomy 7 (10.8%), trisomy 8 (10.4%), and mixed lineage leukemia (*MLL*) rearrangements (8.5%) were the commonest abnormalities [1]. Another study from Canada that analyzed 331 cases of* de novo* AML (101 with extramedullary involvement and 230 without) showed that t(8:21) was present in only 3.9% of extramedullary cases and 5.7% of cases without extramedullary involvement, a difference that was statistically insignificant [21]. The association of MS with t(8:21) may have been biased by analyses of large pediatric cohorts; in such cases, orbital involvement was the most common presentation [1, 20]. In adults, t(8:21) was more commonly associated with paraspinal involvement [20]. In the Canadian study, the only cytogenetic abnormality with a statistically significant different incidence between the groups was the 11q23 abnormality (11.7% versus 2.1%). Other notable differences between the groups included a higher incidence of AML M4 (36.6% versus 25.6%), AML M5 (18.8% versus 9.6%), CD56 expression (22.3% versus 9.9%), and leukocytosis (28,100/*μ*L versus 9,500/*μ*L) in cases with extramedullary involvement. The Italian study had shown CD68/KP1 to be the most commonly expressed marker (100%), followed by myeloperoxidase (83.6%), CD117 (80.4%), CD99 (54.3%), CD68/PG-M1 (51%), CD34 (43.4%), terminal-deoxynucleotidyl-transferase (31.5%), CD56 (13%), CD61/linker for activation of T cells (2.2%), CD30 (2.2%), and CD4 (1.1%). Taken together, key observations from the two studies mentioned above include the association of 11q23 abnormalities, CD56 expression, and AML with myelomonocytic/monoblastic differentiation (M4/M5a) with MS formation. A retrospective study showed that 17 out of 20 cases of MS associated with inv(16), the other CBF abnormality in AML, had abdominal involvement with intestine being the most common site (13 of 17 cases) [22]. Unfortunately, inadequate tissue for analysis precluded cytogenetic or molecular profiling of our patient's MS.

MS has traditionally been considered a poor risk factor in AML. Whether it truly confers a poor prognosis is, however, uncertain. A retrospective study showed a nonsignificantly increased two-year event free (32% versus 18%) and overall survival (OS; 43% versus 29%) in isolated MS as compared to leukemic AML [23]. The optimal treatment of isolated MS is unclear, given the rarity of the diagnosis, variability in presentation, and the lack of prospective studies. A retrospective study evaluated the time to a diagnosis of leukemic AML in patients diagnosed with isolated MS treated with surgical, radiation, or systemic chemotherapy and showed that this time period was significantly longer in patients treated with systemic chemotherapy (median 3 months, 6 months, and 12 months, resp.) [16]. Similarly, another retrospective study showed that early initiation of antileukemic chemotherapy was associated with significantly lower probability of developing leukemic AML (41% versus 71%) and with longer survival (>50% alive at a median follow-up of 25 months compared with median survival of 13 months for those initially untreated) [24]. Treatment of isolated MS similar to leukemic AML with induction chemotherapy is now standard. It is notable that isolated MS almost always proceeds to frank leukemia, although cases without progression even upon long-term follow-up have been reported [6, 25]. It is possible that subclinical involvement of the bone marrow, as documented in rare cases using RT-PCR for gene fusion transcripts, contributes to disease progression in patients treated with local therapies only [26, 27]. However, this has not been clearly established. Currently, surgical and radiation therapy are accepted treatment modalities; however, their precise roles in the treatment algorithm are not well defined [8]. Rapid symptomatic relief, initial debulking, inadequate response to chemotherapy, and recurrence after hematopoietic stem cell transplantation (HSCT) are some of the indications for these ancillary therapeutic modalities [8]. Nevertheless, the effectiveness of these therapies in addition to induction chemotherapy as compared to chemotherapy alone is unknown [8, 24, 28]. The role of allogeneic HSCT was evaluated in a retrospective cohort study involving 99 patients [29]. The study showed no differences in 5-year leukemia-free and OS rates between the isolated and leukemic MS groups. The 5-year leukemia-free and OS were 36% and 48%, respectively, for the entire cohort, thus favoring allogeneic HSCT. Results from the aforementioned Italian study corroborate this finding [1]. Patients treated with HSCT were more frequently long-term survivors, whereas those who received conventional therapies most often rapidly died of their disease (OS at 48 months: 76 versus 0%) [1]. Even those who died after transplant had better survival (mean: 41 months) than those who underwent conventional chemotherapy, imatinib, surgery, or radiotherapy (mean: 7.1 months, 5.6 months, 36 days, and one week, resp.) [1]. Although a retrospective study from a Taiwanese institution did not show any difference in prognosis between patients who underwent allogeneic HSCT and those who did not, the result could have been affected by the small number (4) of patients who underwent HSCT [6]. Several excellent reviews have addressed the management of MS [8, 30, 31].

## 4. Conclusion

Isolated MS is an extremely rare disease and isolated gastric MS even more so. Like all isolated MS cases, it poses diagnostic challenges, given its rarity. Clinical suspicion is the key to correct diagnosis. Morphologic evaluation, along with IHC, flow cytometry, cytogenetics, and molecular studies should be performed and systemic treatment for AML, with or without local therapies, pursued.
